# Differential Energy Criterion for Brittle Fracture: Conceptualization and Application to the Analysis of Axial and Lateral Deformation in Uniaxial Compression of Rocks

**DOI:** 10.3390/ma16134875

**Published:** 2023-07-07

**Authors:** Vitali Shekov, Gennady Kolesnikov

**Affiliations:** Institute of Geology, Karelian Research Centre, Russian Academy of Sciences, Pushkinskaya St. 11, IG KarRC RAS, 185610 Petrozavodsk, Russia

**Keywords:** fracture mechanics, brittle materials, complete stress–strain curve, differential energy fracture criterion, analytical model, graphical model

## Abstract

This paper discusses modeling the behavior and prediction of fracture of brittle materials. Numerous publications show that progress in this area is characterized by the emergence of a number of new models that meet the requirements of the mining industry, construction and other engineering practices. The authors focus only on one class of models, paying special attention to the compromise between simplicity of solution and versatility of the model. A new version of the model is proposed, taking into account the advantages of previous models. We present a differential energy criterion for brittle fracture substantiated, according to which, fracture occurs at a certain ratio of dissipated and stored (elastic) energy. Fracture is considered as the end of the deformation process with a virtual transformation of the initial material almost without cracks into a real material with cracks. The highest and lowest elastic moduli are analytically determined, respectively, on the ascending and descending branches of the stress–strain curve. A graphical version of the algorithm for determining the brittle fracture point on the post-peak branch of the stress–strain curve is proposed. The modeling results are consistent with the experimental data known from the literature.

## 1. Introduction

Rocks consist of many small mineral grains that form a strong but brittle framework in which intergranular boundaries, pores, cracks and joints are inevitable due to the conditions of origin and life cycle [1,2,3]. From the point of view of mechanics, such an inhomogeneous structure is characterized by a nonlinear load–displacement dependence. This dependence is an external manifestation of the internal processes of interaction between the aforementioned grains and material strains, i.e., this dependence indirectly reflects all stages of rock behavior as a brittle material and is therefore of paramount importance for predicting the strength and fracture of rocks and similar materials [4,5,6,7]. Despite progress in this area [8], many criteria have limited application because they do not fully correspond to real materials. At the same time, the best results are obtained using energy criteria [9,10,11].

We focus on the application of the energy approach in combination with Equivalent Material Concept (EMC) [12], which developed in Fictitious Material Concept (FMC) [13].

Note that EMC and FMC are effectively used mainly for modeling load-bearing elements with cracks and notches of various shapes [12,13,14]. Both concepts are based on the idea of replacing a real elastic–plastic material with a model of an ideal brittle material for subsequent application of known and simpler methods of linear analysis. However, the ways of implementing this fundamental idea are different: EMC and FMC focus on the same basic problem related to estimating the critical load on an elastic–plastic element with a notch (or crack) without directly performing a nonlinear analysis, which is complex and time-consuming [15,16]. The EMC replaces the nonlinear load–displacement relation of the real material with a linear (virtual) relation with an appropriate equivalent strength and preserves the same elastic modulus as the real material [14]. Another approach, according to FMC, is that the real and virtual material have different values of the modulus of elasticity, but the strain at the maximum stress is the same. This means that the ultimate strength of the fictitious material may differ from the ultimate strength of the real material. The essential difference between EMC and FMC is that EMC assumes the same modulus of elasticity for a real ductile material and a fictitious brittle material, but the strain at failure must be different [14].

The use of EMC-based models has shown to be effective for small notch radii (e.g., 0.15 mm), but predictions lose accuracy if the notch radius increases [15]. A model based on the concept of fictitious material [13] fills this gap.

As the literature review has shown, the development of strength and fracture criteria that take into account the influence of all material defects requires further research, which is especially important for rocks because their physical and mechanical properties are very variable [17,18]. Given that, in general, all models are to some extent approximate [19], and their areas of effective application may not coincide, it is advisable to complement the many known approaches and models of brittle fracture in order to reduce gaps in this area and improve understanding of the behavior of rocks and similar materials for sustainable development. Given this circumstance and the relevance of the problem [20,21], it is relevant to note that, along with the EMC and FMC mentioned above, a third approach is possible, in which the values of both the elastic modulus and strain of the virtual material are equal to the corresponding values for the real material, as briefly discussed in [22]. Conceptualizing and expanding the scope of this approach to model to not only axial, but also lateral deformation and brittle fracture in uniaxial compression is the goal of this paper, which will provide additional tools for analyzing the behavior and predicting the fracture of rocks and similar materials to improve the efficiency and safety of their use for sustainable development.

To make the following text easier to read, we have divided it into small sections, which are interconnected by the logic of the study. In addition, each important point of the study is explained by an example and comparison with the literature modeling results of some rocks. Thus, the logic of the study predetermined the form of its presentation in this article. The conceptual and methodological aspects of the study determine the way to achieve the goal, so these aspects are logically inseparable from the results. Nevertheless, the article is structured, and the above aspects and results of the study presented in separate, but logically related to each other small sections in order, as mentioned above, to facilitate the reading of the research material.

## 2. Conceptual Aspects, Methodology and Results

### 2.1. Preliminary Remarks

As noted above, from the point of view of material mechanics, the heterogeneous structure of rock and similar materials is characterized by a nonlinear load–displacement relation. This dependence is an external manifestation of the internal processes of interaction of the aforementioned grains at the micro-, meso- and macro-levels, i.e., this dependence indirectly reflects all stages of rock behavior as a brittle material and is therefore of paramount importance for predicting the strength and fracture of rocks and similar materials [4,5,6,7]. To model the behavior of such materials, we will use the basic ideas of fracture mechanics [23,24], in which the key role in justifying the analytical load–displacement relation is played by the choice of the damage variable (D), which determines the fraction of damaged material depending on its deformed state. Consider the process of monotonic load growth.

From the physical point of view, it is clear that under monotonic load, as the weakest particles and connections between them are gradually withdrawn from work, the cross-sectional area decreases and the load redistributed to those particles, which are still undestroyed. Therefore, the average stress in the undestroyed particles increases, leading to increasing deformations of the particles in accordance with Hooke’s law. This process is described in terms of weak link theory [25].

**Figure 1:**
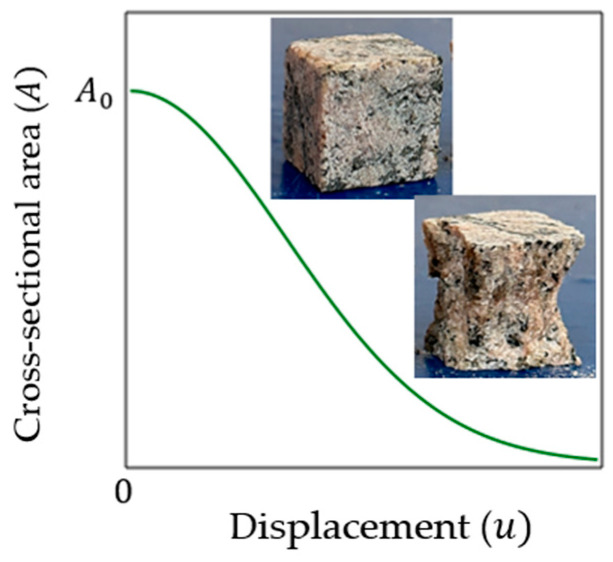
Typical plot of cross-sectional area A versus displacement u under uniaxial compression.

Disconnection of particles and destruction of bonds between them means reduction in the cross-sectional area during loading relative to the initial value ($A_{0}$), so the cross-sectional area (A) should be considered as a variable, which can be related to $A_{0}$ by means of damage variable D. However, to obtain a specific relationship between A and $A_{0}$, it is necessary to use some physically adequate assumption (hypothesis) about the influencing factors. Such a factor can be a change in the characteristic size of a brittle material specimen under uniaxial compression. For example, if the initial height of the specimen is equal to $H_{0}$, and the displacement in the direction of this load is equal to u, then the value of u can be considered an influencing factor, since it is related (according to Hooke’s law) to the internal force in this element. A typical plot of the dependence of the cross-sectional area A on the displacement u shown in Figure 1.

### 2.2. Research Hypothesis

In this section, we consider the problem from a physical point of view, and then formalize the physical model using methods of mathematical modeling.

Based on the ideas of fracture mechanics, we take into account only the effect of material damage on the change in the cross-sectional area of the specimen (Figure 1). To the physical meaning of the problem does not contradict the hypothesis: damage (dA) is proportional to the undamaged part of the area and the power function of displacement (deformation):

(1) $$dA=-A{\left(\frac{u}{u_{peak}}\right)}^{n-1}du.$$

**Figure 2:**
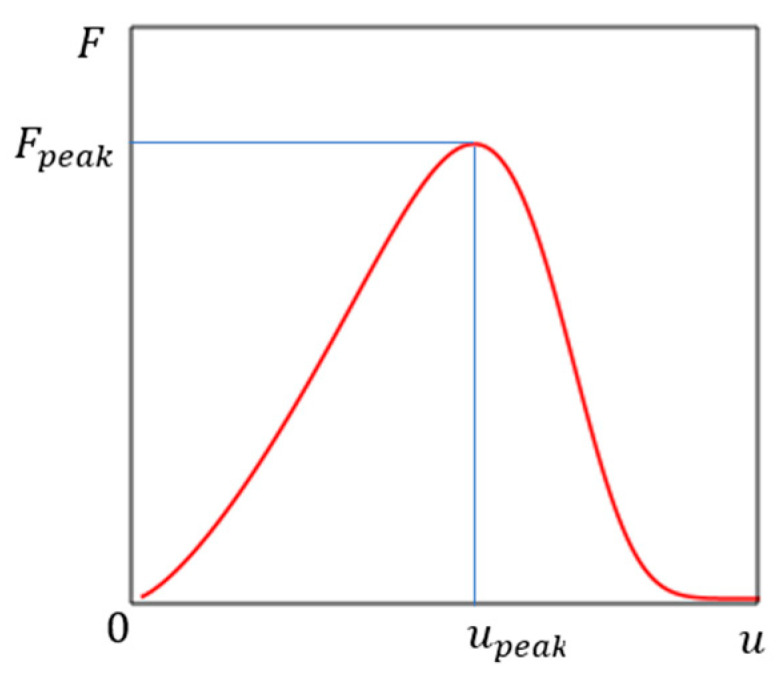
Load vs. displacement plot.

Here the sign “minus” is written, since the function $A\left(u\right)$ is a decreasing function according to the physical meaning of the problem (Figure 1). The initial cross-sectional area is equal to $A_{0}$ (at u=0). The $u_{peak}$ equal to the displacement u at the peak point on the load–displacement graph $F\left(u\right)$ (Figure 2). The model parameter n used to simulate nonlinearity, and determined during model calibration.

Formalizing the hypothesis and considering the problem in terms of mathematical modeling, let us move to dimensionless variables. Let us divide both parts of Equation (1) by $A_{0}$, denote by $\theta =A/A_{0}$ and transform Equation (1) to the form (2):

(2) $$\frac{d\theta }{\theta }=-{\left(\frac{u}{u_{peak}}\right)}^{n-1}du.$$

Given that θ=1 at u=0, we define the function θ from Equation (2) and express A:

(3) $$\theta =e^{-\frac{1}{n}{\left(\frac{u}{u_{peak}}\right)}^{n}};$$

(4) $$A=A_{0}\theta .$$

It follows from relation (4) that the function *θ* defines the undamaged part of the original cross-sectional area; in physical terms, this function called a residual area function (or residual lifetime function). After the residual life function (3), the damage measure (variable) defined as D:

(5) D=1−θ.

Note that various approaches to the definition of D as a measure of material damage under load are well known [26,27,28,29]; usually, the corresponding mathematical expressions are postulated, i.e., accepted a priori or at the level of hypotheses. At the conceptual level, we have tried to minimize the volume of hypotheses, realizing that it is not possible to abandon them completely. Nevertheless, we have considered one-step prior to justifying the magnitude of the damage (5). This small step toward a better understanding of the mechanical behavior of brittle materials is justified by Equations (1)–(4), the application of which discussed below.

### 2.3. Estimates of the Undamaged and Damaged Area Depending on the Strain

**Figure 3:**
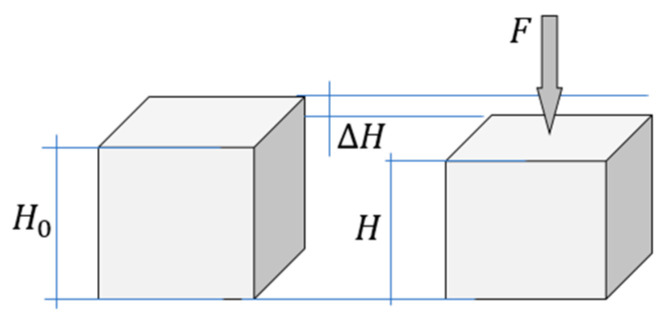
Force F and deformation∆H=u.

Equations (3)–(5) express estimates of the undamaged and damaged portions of the cross-sectional area using displacement *u*. The estimates thus calculated refer to a specific specimen or to a specific structure (Figure 3).

In Equation (3), in order to characterize not only the structure but also the material, relative strain (ε) should be used [30]. If the height of the specimen is equal to $H_{0}$, then $\varepsilon =u/H_{0}$; $u=\varepsilon H_{0}$; $u_{peak}=\varepsilon_{peak}H_{0}$. After substituting the expressions u and $u_{peak}$ in (3) we obtain:

(6) $$\theta =e^{-\frac{1}{n}{\left(\frac{\varepsilon }{\varepsilon_{peak}}\right)}^{n}}.$$

The model parameter n, as noted above, is determined at the stage of model calibration. The value of the undamaged area is equal to (4). The damage measure is determined similarly to (5): D=1−θ.

### 2.4. Degradation of the Cross-Sectional Area

Using the above Figure 3 and relations (4) and (6), consider strain and stress in the damaged material. According to Hooke’s law

(7) $$∆H=u=\frac{FH_{0}}{EA}=\frac{FH_{0}}{EA_{0}\theta }.$$

Using Equation (7), let us define the strain:

(8) $$\varepsilon =\frac{∆H}{H_{0}}=\frac{u}{H_{0}}=\frac{F}{EA}=\frac{F}{EA_{0}\theta }.$$

Under uniaxial load σ=εE. Taking into account relation (8) we write:

(9) $$\sigma =\varepsilon E=\frac{F}{A_{0}\theta }.$$

Ratio (9) shows that the deformation of brittle material degrades the cross-sectional area (Figure 1).

To continue the study, it is necessary to determine the dependence of force F on displacementu; the dependence of stress σ on strainε; and the parameter n in relations (3) and (6).

### 2.5. Justification of the Load–Displacement Relation in Uniaxial Compression

On the conceptual level, we assume that the average value of the elastic modulus of each individual particle, of which a brittle material consists, does not change under loading under normal conditions. This assumption does not contradict modern ideas about materials [30]. This does not deny that the modulus of elasticity (stiffness) of the conglomerate of these particles changes under load.

Using one of relations (7), let us express the modulus of elasticity from it and using Equation (3), we write:

(10) $$u=\frac{FH_{0}}{EA_{0}\theta };E=\frac{FH_{0}}{uA_{0}\theta }=\frac{FH_{0}}{uA_{0}}e^{\frac{1}{n}{\left(\frac{u}{u_{peak}}\right)}^{n}}.$$

Equation (10) is acceptable for any pair of values of u and F corresponding to the physical meaning of the problem, for example, for $u=u_{peak}$ and $F=F_{peak}$:

(11) $$E=\frac{F_{peak}H_{0}}{u_{peak}A_{0}}e^{\frac{1}{n}}.$$

The values of the modulus of elasticity in relations (10) and (11) are the same, which makes it possible to obtain the equation for determining the dependence of the load F on the displacement u:

(12) $$\frac{FH_{0}}{uA_{0}}e^{\frac{1}{n}{\left(\frac{u}{u_{peak}}\right)}^{n}}=\frac{F_{peak}H_{0}}{u_{peak}A_{0}}e^{\frac{1}{n}}.$$

(13) $$F=F_{peak}\frac{u}{u_{peak}}e^{\frac{1}{n}(1-\frac{u^{n}}{u_{peak}^{n}})}.$$

The model parameter n is determined at the stage of model calibration, by analogy with [6].

### 2.6. Justification of the Stress–Strain Relation in Uniaxial Compression

In Equation (13), to characterize not only the structure but also the material, stress (σ) and strain (ε) should be used [30]. As usual, $\sigma_{peak}=F_{peak}/A_{0}$, i.e., $F=\sigma A_{0},F_{peak}=\sigma_{peak}A_{0}$. If the height of the specimen is equal to $H_{0}$ (Figure 3), then $\varepsilon =u/H_{0}$; $u=\varepsilon H_{0}$; $u_{peak}=\varepsilon_{peak}H_{0}$. After substituting these expressions F,u and $u_{peak}$ in Equation (13) we obtain:

(14) $$\sigma =\sigma_{peak}\frac{\varepsilon }{\varepsilon_{peak}}e^{\frac{1}{n}(1-\frac{\varepsilon^{n}}{\varepsilon_{peak}^{n}})}.$$

As noted above, the model parameter n is determined during model calibration, by analogy with [6].

### 2.7. Previous Models of the Class under Discussion

We consider models belonging to the same class, which begins with the Furamura model (15) [6]:

(15) $$\sigma =\sigma_{peak}\frac{\varepsilon }{\varepsilon_{peak}}e^{1-\frac{\varepsilon }{\varepsilon_{peak}}}.$$

A comparison of Equations (14) and (15) shows that if n=1, we obtain a simpler model (15). Thus, our proposed Equation (14) is a generalization of model (15), which extends the scope of its application.

We obtain more opportunities for nonlinear analysis if we use the Blagojevich model (16) [31]:

(16) $$\sigma =\sigma_{peak}{\left(\frac{\varepsilon }{\varepsilon_{peak}}e^{1-\frac{\varepsilon }{\varepsilon_{peak}}}\right)}^{B}.$$

DDThe model parameter B is determined when calibrating the model, for example, using the least-squares method [6].

By analogy with (16), we can modify model (14) to the form (17):

(17) $$\sigma =\sigma_{peak}{\left(\frac{\varepsilon }{\varepsilon_{peak}}e^{\frac{1}{n}(1-\frac{\varepsilon^{n}}{\varepsilon_{peak}^{n}})}\right)}^{B}.$$

Thus, model (14) and a modification of this model (17) represent two new steps in the progress of models of this class. The universality of models (14)–(17) is explained by the possibility of using them to independently control the pre-peak and post-peak branches of the full load–displacement (stress–strain) curve [6,32]. The parameters B and n specified in (17) can be integers and fractions.

Note also that Equations (14)–(17) are indifferent to signs of deformations, so they can be used for both compression and tension. Analysis of accumulated energy can be performed by integrating these equations. Thus, application of the analytical model (17) can improve understanding of the mechanical behavior of brittle materials, such as clarifying notions of the tangential modulus of elasticity in the pre-peak and post-peak stages, as discussed below.

### 2.8. Pre- and Post-Peak Modulus of Elasticity

Analysis of brittle material state changes under its loading is a part of many research studies, the review and the current state of which can found in [33]. Besides the review, in [33], experimental data and analysis of tests of marble and granite on test machines of different stiffness are given. These results are a good basis for checking the adequacy of model (17) as a tool for analyzing the mechanical behavior of brittle material. We focus on the analysis of changes in the modulus of elasticity.

Using the second Equation (17), we define a function that models the dependence of elastic modulus on strain:

(18) $$E\left(\varepsilon \right)=\frac{d\sigma }{d\varepsilon }=\frac{\sigma_{peak}}{\varepsilon }(1-\frac{\varepsilon^{n}}{\varepsilon_{peak}^{n}})B{\left(\frac{\varepsilon }{\varepsilon_{peak}}e^{\frac{1}{n}(1-\frac{\varepsilon^{n}}{\varepsilon_{peak}^{n}})}\right)}^{B}.$$

The value of ε, at which the extremum of the function (18) is reached, can be found from the condition (19):

(19) $$\frac{d^{2}\sigma }{d\varepsilon^{2}}=0.$$

Using (19), we obtain two values of ${\varepsilon =\varepsilon }^{left}$ and $\varepsilon =\varepsilon^{right}$, for the left and right branches, respectively:

(20) $$\varepsilon^{left}=\varepsilon_{peak}{\left(1+\frac{n-1-\sqrt{4Bn+{\left(n-1\right)}^{2}}}{2B}\right)}^{\frac{1}{n}};$$

(21) $$\varepsilon^{right}=\varepsilon_{peak}{\left(1+\frac{n-1+\sqrt{4Bn+{\left(n-1\right)}^{2}}}{2B}\right)}^{\frac{1}{n}}.$$

Substituting $\varepsilon =\varepsilon^{left}$ and ${\varepsilon =\varepsilon }^{right}$ in Equation (18), we calculate, respectively, tangential modulus of elasticity *E* and post-peak modulus M (review and new results related to post-peak modulus are given in [33]). In the following section, we consider an example of the application of Equations (18)–(21) and compare it with the experimental data known from the literature.

### 2.9. Comparison of Simulation Results and Experimental Data

In this section, we will compare the results obtained with the proposed model (17) and experimental data for uniaxial compression of marble from [33] for two relations: axial stress–axial strain and axial stress–lateral strain. The same model (17) is used to analyze both axial and lateral deformations, but with different values of model parameters. Initial data for calculations using Equation (17) given in Table 1 and Table 2.

**Figure 4:**
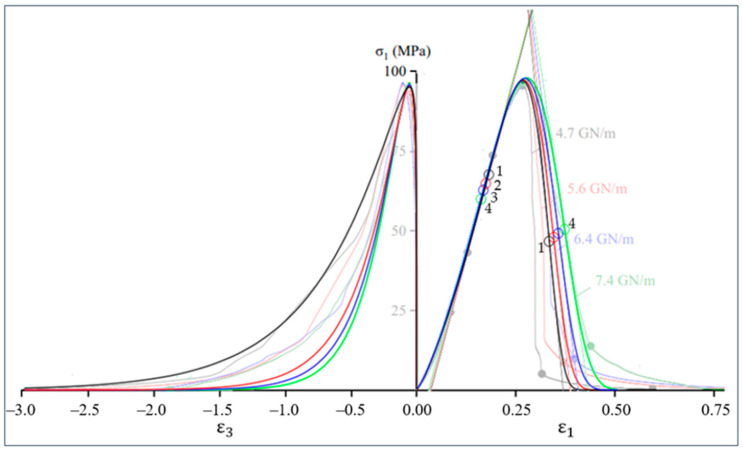
Plots of axial stress–axial strain and axial stress–lateral strain during uniaxial compression of marble. The semi-transparent color shows experimental data from [33] obtained with test machines of different stiffness (4.7, 5.6, 6.4 and 7.4 GN/m). Colored circles 1, 2, 3 and 4 correspond to the points at which the condition (19) is satisfied; tangents (thin slanted lines) pass through these points. The tangent angle of these lines determines the pre- and post-peak moduli (Table 3).

The Poisson’s ratio used in Table 2 to determineε peak using data from Table 1, e.g., $\varepsilon_{peak}=-0.2\cdot 0.268=-0.0536$. The simulation results shown in Figure 4 and Table 3.

Figure 4 and the first column of Table 3 show that model (17) is suitable for analyzing the behavior of brittle material at the pre-peak stage of deformation. The post-peak modulus is considered in detail in [33]. Note that according to [33], the values of the post-peak modulus significantly depend on the stiffness of the test machine and, for example, during uniaxial compression of marble, these values can vary in the range from −550 to −0.49 GPa. To improve the accuracy of modeling the post-peak stage of axial deformation, it is reasonable to use independent control of the parameters of the pre-peak and post-peak branches of the full stress–strain curve [32]; details of this issue are beyond the scope of this paper.

### 2.10. The Point of Maximum Modulus of Elasticity on the Ascending Branch of the Stress–Strain Curve as the Point of Highest Density of a Brittle Material

At the pre-peak stage of deformation (Figure 4), the initial concave stress–strain curve simulates the stage of cracks closure, in which a denser packing of material particles formed, leading to a certain decrease in porosity and an increase in material density. Therefore, the modulus of elasticity increases, which is confirmed by the increase in the angle of inclination of the tangent, i.e., the growth of the modulus of elasticity to the maximum values at points 1, 2, 3 and 4 (Figure 4). If we assume that there were no cracks at the beginning of the material life cycle, then the highest value of the modulus may be considered as an estimate of the modulus of elasticity of the native material.

Since the real material is inhomogeneous and has some plasticity potential, the load growth is accompanied by the interaction of two opposite tendencies, one of which is compaction and growth of elastic modulus and the other is growth of plastic deformations and gradual disconnection of the weakest links (in accordance with the theory of a weak link [25]). The interaction of these trends leads to the appearance of an inflection point on the ascending branch. For the specimens 1, 2, 3 and 4 (Table 1, Table 2 and Table 3) these points are marked by colored circles with the same numbers in Figure 4, which shows that after passing through these points the material destruction intensifies.

The inflection points on the descending branch of the stress–strain curve for specimens 1, 2, 3 and 4 also indicated in Figure 4.

The points with abscissa (20) and (21) determine, as indicated above, the largest pre-peak and smallest post-peak moduli (Table 3). Thus, the points for which condition (19) is satisfied can referred to characteristic points on the stress–strain curve. The practical value of these modules lies, for example, in their application in justification of strength and fracture criteria for brittle materials [22].

### 2.11. The Concept of Virtual Material Transformation

**Figure 5:**
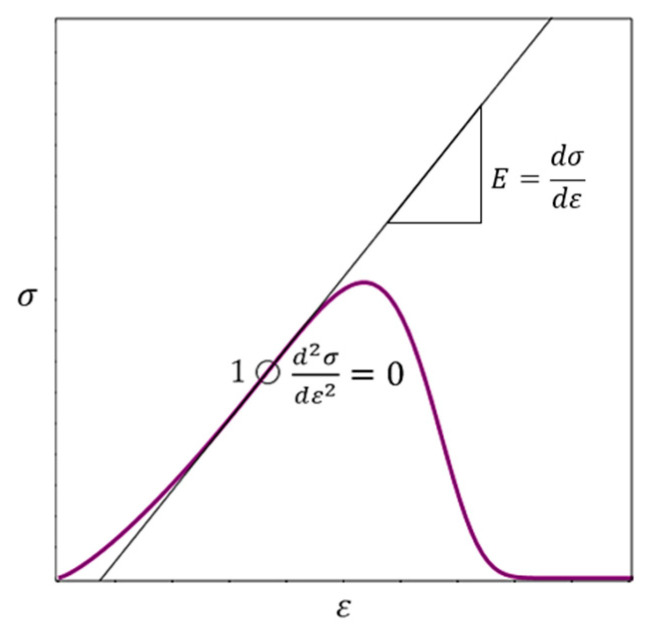
Axial stress–axial strain relationships for an ideal material (thin straight line) and a real material (curve).

Virtual transformation means the transition from the native material as it was at birth to the real cracked worn material. The observation mentioned in Section 2.10 that the highest value of the elasticity modulus for a real material with cracks closed in compression considered as an estimate of the elasticity modulus for a native material induces the idea of a virtual transformation of an initially ideal elastic material to a real material with cracks. The stress–strain relationship for the ideal and real material shown in Figure 5 as a straight line and a curved line, respectively.

The tangent to the curve in Figure 5 passes through point 1 for which condition (19) holds. The abscissa of point 1 can be calculated by Formula (20), then calculate the highest value of the modulus of elasticity (18).

**Figure 6:**
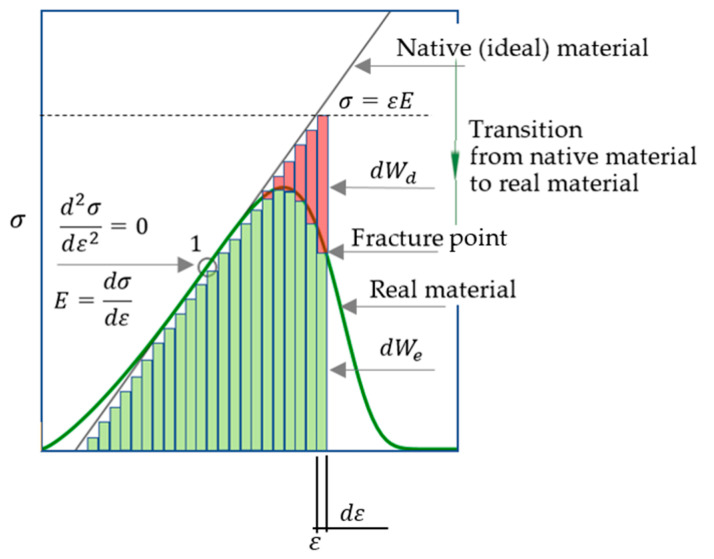
Change in dissipated ($dW_{d}$) and stored ($dW_{e}$) energy at infinitesimal increment of deformation (dε). The modulus of elasticity is greatest at point 1. Point 1 can be determined numerically (by processing measurements during material tests) or analytically (by differentiating the function (17)); see also Section 2.8. The stress–strain relation (σ=εE) for the ideal material is represented by the tangent to the stress–strain curve (17) for the real material; point 1 is the common point of these dependencies, i.e., as noted above, the elastic modulus of the ideal material at this point is equal to that of the real material.

In this paper, we consider the virtual transformation from an ideal material to a real material as a long-term process of summation of a very large number of small damages arising from external influences. Under load, conglomerates of material particles deform and internal forces arise in them. If these forces are large enough, the weakest particles and particle connections gradually damaged and disconnected (partially or completely). For this reason, part of the input energy is dissipated. Another part of the input energy is stored in the undamaged deformed particles. The energy calculated as the area under the stress–strain curve, i.e., an integral approach is used. Note that such ideas about energy consumption during deformation of elastic–plastic and brittle materials are basic in a large number of studies [34,35,36,37,38,39,40,41]. We will consider the ratio of stored ($dW_{e}$) and dissipated ($dW_{d}$) energy at an infinitesimal change in material strain (*dε*) at the final stage of brittle fracture, i.e., we will use the differential approach to determine the “last straw” (Figure 6).

The issue of the coordinates (ε and σ) of the fracture point (Figure 6), at the level of idea, briefly discussed in [22]. This issue is key in the context of the current study, so it investigated in more detail, using Equation (17) in Section 2.12.

### 2.12. Relationship between $dW_{e}$ and $dW_{d}$, Strength Condition and Fracture Point Coordinates

Figure 6 shows that the fracture point is on the stress–strain curve for the real material. The following equations can written:

(22) $$dW_{e}+dW_{d}=dW=\sigma d\varepsilon =\varepsilon Ed\varepsilon .$$

To determine the coordinates ε and σ of the fracture point (Figure 6), two equations needed. The stress–strain curve Equation (17) is one of these equations. The second equation can be obtained by finding the relationship between $dW_{e}$ and $dW_{d}$. This requires answering two questions [22].

Question 1: If ε>0, is equality $dW_{d}=0$ possible?

Answer: Yes, it is possible. Equality $dW_{d}=0$ is realized, for example, at point 1 (Figure 6).

Question 2: If ε>0, is the equality $dW_{e}=0$ possible?

Answer: If ε>0, the equality $dW_{e}=0$ is impossible. Indeed, if ε>0, then $dW=dW_{e}+dW_{d}=0+dW_{d}=dW_{d}$. From the physical point of view, the equality $dW=dW_{d}$ means that the input energy is completely dissipated and σ=0, i.e., the material is nonfunctional. From a physical point of view, for real materials, if ε>0, then $dW_{e}>0$.

From the answers to questions 1 and 2 it follows: if ε > 0, then the inequality (19) is satisfied for real brittle materials:

(23) $$dW_{e}>dW_{d}.$$

It follows from (22):

(24) $$dW_{d}=dW-dW_{e}.$$

Let us substitute $dW_{d}$ (24) into inequality (23). We obtain:

(25) $$dW_{e}>\frac{dW}{2}.$$

From a physical point of view, the material is functional, i.e., the material resists the load if inequality (25) fulfilled. Therefore, inequality (25) used to construct a differential criterion for the strength of a brittle material [22].

If strict inequality (25) is a condition of strength, then the condition of destruction is a non-strict inequality (26):

(26) $$dW_{e}\leq \frac{dW}{2}.$$

The non-strict inequality (26) contains two conditions: $dW_{e}<dW/2$ and $dW_{e}=dW/2$. It is necessary to answer the question: which of these conditions corresponds to the physical meaning of the problem? Since dW=εEdε (18), then instead of (26) we can write (27):

(27) $$dW_{e}\leq \frac{\varepsilon Ed\varepsilon }{2}.$$

According to the physical meaning of the problem, the fracture point on the descending branch of the stress–strain curve corresponds to the smallest value of ε (Figure 6). In this case, the non-strict inequality (27) realized in the form of equality (28):

(28) $$dW_{e}=\frac{\varepsilon Ed\varepsilon }{2}.$$

Given equality (22) and the failure condition (28), we obtain the following equations:

(29) $$dW_{e}=\frac{dW}{2}=\frac{dW_{e}+dW_{d}}{2}or\frac{{dW}_{e}}{2}=\frac{dW_{d}}{2};{dW}_{e}=dW_{d}.$$

The stress and strain at the fracture point (Figure 6) are denoted, respectively, as $\sigma_{fracture}$ and $\varepsilon_{fracture}$. Given Equation (22), we rewrite Equation (29) as (30), then (31) and (32):

(30) $$\sigma d\varepsilon =\frac{\varepsilon Ed\varepsilon }{2};$$

(31) $$\sigma =\frac{\varepsilon E}{2};$$

(32) $$\sigma_{fracture}=\frac{\varepsilon_{fracture}E}{2}.$$

Using relation (17), we write the left part of Equation (32) in the form (33):

(33) $$\sigma_{fracture}=\sigma_{peak}{\left(\frac{\varepsilon_{fracture}}{\varepsilon_{peak}}e^{\frac{1}{n}(1-\frac{\varepsilon_{fracture}^{n}}{\varepsilon_{peak}^{n}})}\right)}^{B}.$$

Finally, using (32) and (33), we obtain Equation (34) to calculate $\varepsilon_{fracture}$:

(34) $$\sigma_{peak}{\left(\frac{\varepsilon_{fracture}}{\varepsilon_{peak}}e^{\frac{1}{n}(1-\frac{\varepsilon_{fracture}^{n}}{\varepsilon_{peak}^{n}})}\right)}^{B}=\frac{\varepsilon_{fracture}E}{2}.$$

**Figure 7:**
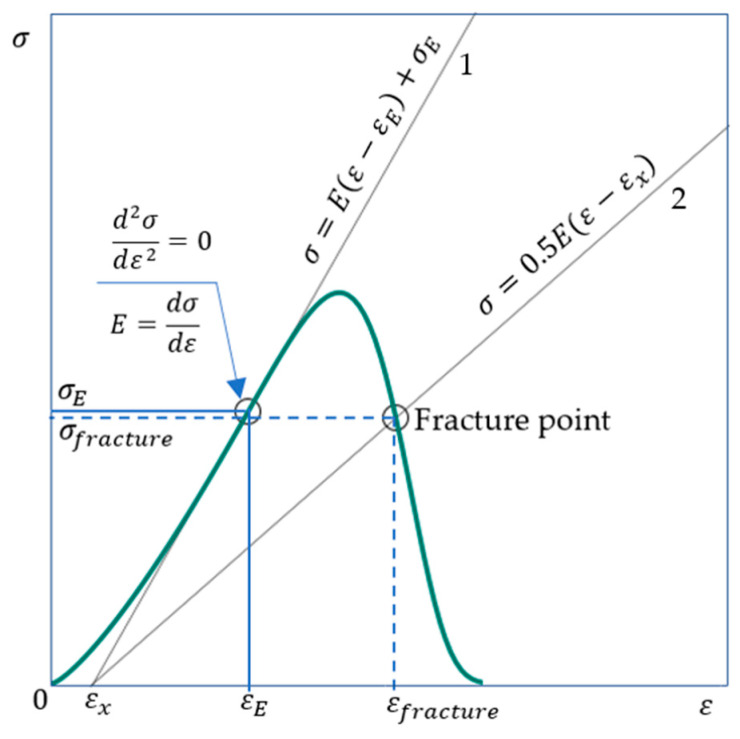
The coordinates of the fracture point $\varepsilon_{fracture}$ and $\sigma_{fracture}$ rupture are determined from Equations (34) and (33), respectively. Lines 1 and 2 intersect at the point with coordinates $\varepsilon_{x}=\varepsilon_{E}-\frac{\sigma_{E}}{E}$; σ=0. Elastic modulus E calculated from Equations (18) and (20). The point with coordinates $\varepsilon_{E},\sigma_{E}$ corresponds to the highest value of the modulus of elasticity.

The modulus of elasticity (E) can be determined as shown above in Section 2.8; the determination of the parameters n and B is discussed by example in Section 2.9. The peak strain and stress ($\varepsilon_{peak}$ and $\sigma_{peak}$) are determined experimentally, but in some cases these values can be predicted using experimental data only for the pre-peak stage [32,39]. Additional graphical explanations of Equation (34) shown in Figure 7.

### 2.13. Graphic Definition of the Brittle Fracture Point

**Figure 8:**
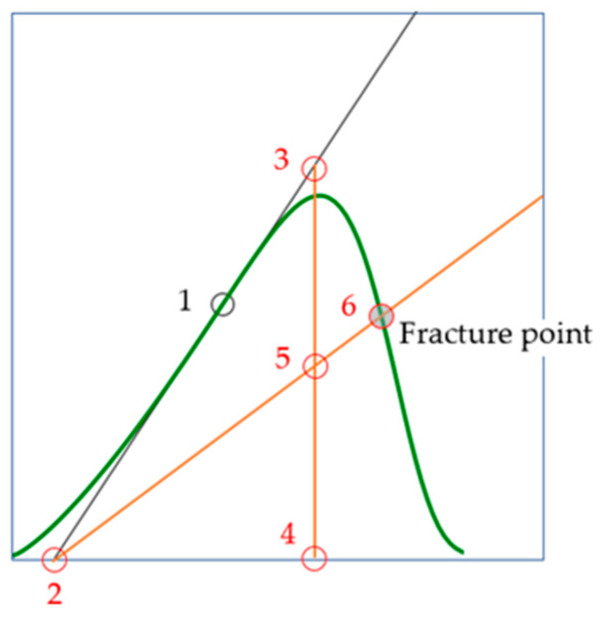
Point 1 corresponds to the highest value of the modulus of elasticity. Point 6 corresponds to brittle fracture. Points 2–4 are auxiliary points.

In the previous section, we considered an analytical model for determining the brittle fracture point. Considering the above, we can propose a simple algorithm for graphically determining the fracture point of a brittle material using a complete stress–strain curve (Figure 8):

1. On the pre-peak branch, determine the point that corresponds to the largest tangential modulus of elasticity (the point 1 in Figure 8).
2. Draw a tangent through point 1 and define point 2.
3. From any point (for example, point 3) on the tangent, draw a perpendicular to the abscissa axis. Find point 4.
4. Define point 5 as the midpoint of segment 3–4.
5. Draw a line through points 2 and 5. The point of intersection of this line with the post-peak branch of the stress–strain curve simulates the point of failure; this is point 6 in Figure 8.

**Figure 9:**
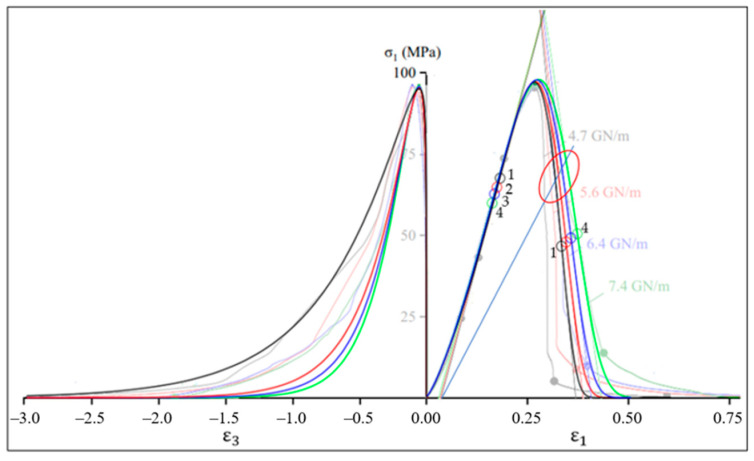
The ellipse limits the area of predicted fracture points on the curves by Figure 4.

An example of applying the graphical determination of predicted fracture points for the four curves by Figure 4 shown in Figure 9.

## 3. Discussion

The main result in this paper is models (14) and (17), which belong to the same class of models as the preceding models known from the literature [6,31,32]. Models (14) and (17) are more versatile as compared to their predecessors, since depending on the parametern in (14) and (17), known models can be obtained. For example, model (14) at n=3 coincides with the model from [34], which are obtained using the energy approach:

(35) $$\sigma =\sigma_{peak}\frac{\varepsilon }{\varepsilon_{peak}}e^{\frac{1}{3}(1-\frac{\varepsilon^{3}}{{\varepsilon ,}_{peak}^{3}})}.$$

If n=1, then model (14) coincides with the Furamura model [6]:

(36) $$\sigma =\sigma_{peak}\frac{\varepsilon }{\varepsilon_{peak}}e^{\begin{array}{c} 1-\frac{\varepsilon }{\varepsilon_{peak}} \end{array}}.$$

**Figure 10:**
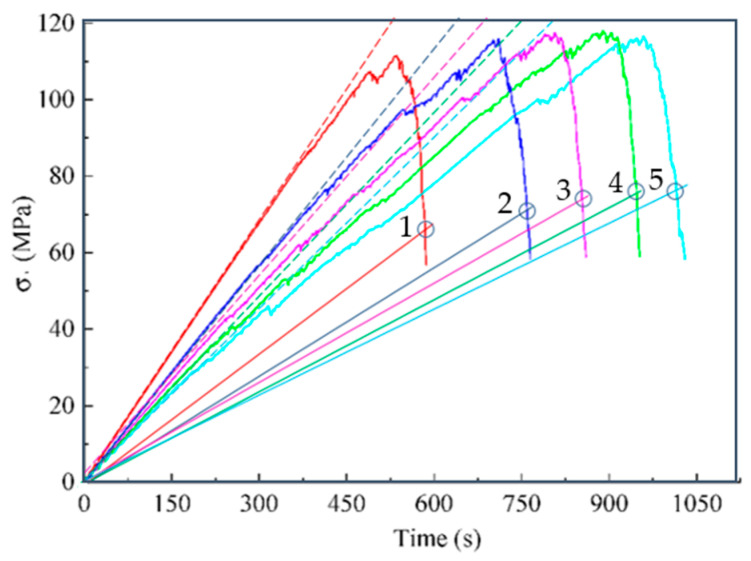
The experimental curves are adapted from [40]; points 1–5 on these curves predict the failure of granite specimens with different modulus of elasticity in uniaxial compression. Points 1–5 obtained as a solution of Equation (34) using the graphical algorithm (Section 2.13).

Thus, the known models explicitly consider only energy aspects or only deformation aspects. Given the heterogeneity of rocks, we can assume that the model will be more accurate if different aspects are taken into account. The proposed models (14) and (17) differ in the fact that the parameter n can be a fractional number (n≥1), which makes it possible to consider in different proportions both energy and deformation aspects, as well as possible other effects in the same equation. For example, 6.0≤n≤9.5 in Table 1 above and 1.00≤n≤1.15 in Table 2. An even more versatile analysis tool in this area is model (17), the results of which (Figure 4 and Table 3) do not contradict experimental data known from the literature for marble [33]. The application of the graphic algorithm for determining the predicted fracture points (Section 2.13) on the experimental curves in uniaxial compression of granite [40] shown in Figure 10.

The considered examples show the consistency of the simulation results with the experimental data.

We considered the issue of analytical determination of the highest value of the modulus of elasticity. Equations (18)–(20) for calculating this modulus were obtained (Table 3), which refer to the main results of the current study, because on their basis we formulated the hypothesis that, at the initial stage of the brittle material life cycle, the elastic modulus estimate is equal to the highest value of the elastic modulus of the real material (Section 2.10). In development of this hypothesis, the concept of virtual material transformation is proposed (Section 2.11) and the approach (Figure 6) for determining the differential-energy criterion for brittle fracture is justified. Equations for calculating strain (34) and stress (33) at the point of brittle fracture derived. In addition, in view of this result, a graphical algorithm for determining the point of brittle fracture proposed (Figure 8). An example of consistency with experimental data known from the literature given (Figure 9). However, the practice of application of these results is insufficient to recommend the practical application of these results, that is, further research needed.

The above may be of interest for future research on this topic. First, the effect of material stiffness and load change rate on model parameters n and B (18) should be investigated. At this point in time, it is clear that the test results depend not only on the stiffness of the specimen, but also on the stiffness of the test machine [33]; however, refinement of these representations requires further research. In addition, it is important to consider the behavior of brittle materials under seismic loading, despite the large number of studies in this area [42,43,44].

## 4. Conclusions

This article considers the approach to justification of the differential energy criterion of rock brittle fracture. It focuses on the conceptualization and application to the analysis of axial and lateral strain during uniaxial compression and fracture of rocks using marble and granite as examples. The study is based on previous results known from the literature.

1. The hypothesis of the study was formulated (1): the damage to the cross-sectional area is proportional to the undamaged part of the area and to displacement (strain).
2. In a logical connection with the research hypothesis (1), the residual resource function of the cross-sectional area (3) is justified.
3. Based on the research hypothesis, new variants of load–displacement models (13) and stress–strain models (14), (17) were justified, taking into account both axial and lateral deformations (Table 1 and Table 2, Figure 4).
4. The question of analytical determination of the highest (pre-peak) and lowest (post-peak) values of the modulus of elasticity is considered. Equations (20) and (21) for calculating the coordinates and values (18) of these moduli are obtained. Examples of determining the pre-peak and post-peak modulus of elasticity in uniaxial compression of marble are given (Table 3).
5. An algorithm for the graphical determination of the brittle fracture point is proposed (Figure 8). An example of prediction based on experimental data known from the literature is given (Figure 9).

Given the small amount of research using the developed models, it is necessary to continue research in this direction, despite the consistency of simulation results and experimental data known from the literature.

## Figures and Tables

**Table 1:** Input data and calibration parameters for modeling axial strain.

Specimen Number	Input Data		Calibration Parameters	
	$\varepsilon_{peak}$ (%)	$\sigma_{peak}$ (MPa)	n	B
1	0.268	95.6	9.5	1.3
2	0.270	95.8	8.0	1.3
3	0.274	96.3	7.0	1.3
4	0.279	96.4	6.0	1.3

**Table 2:** Input data and calibration parameters for modeling lateral strain.

Specimen Number	Input Data			Calibration Parameters	
	Poisson’s Ratio *ν*	$\varepsilon_{peak}$ (%)	$\sigma_{peak}$ (MPa)	n	B
1	0.2	−0.0536	95.6	1.00	0.10
2	0.2	−0.0540	95.8	1.05	0.20
3	0.2	−0.0548	96.3	1.08	0.20
4	0.2	−0.0578	96.4	1.15	0.20

**Table 3:** Pre- and post-peak module by (18)–(20).

Specimen Number	Pre-Peak Module			Post-Peak Module		
	*E* (GPa)	ε (%)	σ (MPa)	M (GPa)	ε (%)	σ (MPa)
1	46.1 (46.8) ^1^	0.183	66.67	−133.34	0.336	45.85
2	45.9 (44.7)	0.175	63.89	−112.48	0.346	47.18
3	45.7 (44.6)	0.169	61.83	−98.05	0.359	48.47
4	45.2 (45.5)	0.163	59.01	−83.21	0.374	49.81

^1^ The experimental values of the elastic modulus from [33] are given in parentheses.

## Data Availability

No new data were created or analyzed in this study. Data sharing is not applicable to this article.

## Nomenclature

A	cross-sectional area of the specimen.
B	model parameter.
D	damage variable.
E	Young’s modulus.
EMC	concept of equivalent material [12].
F	Force (or Load).
FMC	concept of fictitious material [13].
n	model parameter.
u	displacement.
$u_{peak}$	peak displacement.
$W_{d}$	dissipated energy.
$W_{e}$	stored energy.
ε	strain.
$\varepsilon_{fracture}$	strain in fracture point.
$\varepsilon_{peak}$	peak strain.
σ	stress.
$\sigma_{fracture}$	stress in fracture point.
$\sigma_{peak}$	peak stress.
*θ*	undamaged part of the original cross-sectional area.
